# Commentary: Population pharmacokinetics of colistin sulfate in critically ill patients: Exposure and clinical efficacy

**DOI:** 10.3389/fphar.2022.992085

**Published:** 2022-09-09

**Authors:** Huadong Chen, Piaopiao Li

**Affiliations:** Department of Pharmacy, Affiliated Dongyang Hospital of Wenzhou Medical University, Dongyang, China

**Keywords:** polymyxin, colistin, pharmacokinetic, pharmacodynamic, dose optimization

## Introduction

Recently, Yu et al., 2022 published a study of great interest “Population pharmacokinetics of colistin sulfate in critically ill patients: Exposure and clinical efficacy” in Frontiers in Pharmacology. Polymyxins are considered as last-resort for treatment of infection caused by carbapenem resistant organisms (Heavner et al., 2018). Usually, there are two available polymyxin products, polymyxin B sulfate and sodium colistin methanesulfonate. But a new form of polymyxin for intravenous use, colistin sulfate, came back to Chinese market recently. It has a similar chemical structure as polymyxin B but usually does not cause skin hyperpigmentation after intravenous administration (Nahhas et al., 2019). This population pharmacokinetic study of colistin sulfate is important for its optimal use. However, we believe that some weak points in the research should be addressed and this would benefit readers.

## Dose of colistin sulfate

Different conventions are used to define the dose of polymyxins, and this has caused significant confusions in polymyxin dosing (Li et al., 2006; Nation et al., 2015). The product of colistin sulfate is labeled as 500,000 IU per vial. But we used mg/L when we determined the MIC of bacteria or drug concentration in plasma. The insert of colistin sulfate does not provide the convention of mg and IU, and there are few papers about the convention. Yu et al. claimed that according to an expert consensus by the Infectious Disease Society of China, 1 mg of pure colistin base equals 17,000 IU of colistin (Infectious Diseases Society of China, 2021). It is not correct. The expert consensus clearly indicated that 1 mg of colistin sulfate equals approximately 22,300 IU (Infectious Diseases Society of China, 2021). In the Chinese Pharmacopeia, it says that the titer of 1 mg colistin sulfate should be no less than 17,000 IU (Chinese Pharmacopeia Committee, 2015). But it is the low-limit of potency and should not be used for convention. The manufacture should improve the label and convention of colistin sulfate to facilitate its use.

## Pharmacokinetic/pharmacodynamic modeling

Yu et al. had developed a population pharmacokinetic model for colistin sulfate, and the model only accounts for intravenous administration. But 52.4% of the included patients received inhaled colistin sulfate. There is no data on how much of inhaled colistin can reach system circulation. If the amount reaching the systemic circulation was not negligible, then the simulations from the developed population PK model would probably overestimate the plasma concentration given that input from other routes of administration was ignored during model development.

The result of Monte Carlo simulation by Yu et al. indicated that the probability of target attainment (PTA) of PKPD target was satisfied when the minimum inhibitory concentration (MIC) of bacteria was 0.5 mg/L. But for pathogens with an MIC of 1 mg/L, only 3 off-label dosages (the maximum daily dose was 1.5 million IU) had PTA>90% in patients with different renal function levels. However, pathogens with MICs of 0.5 mg/L or below only take a small part. EUCAST MIC distribution data indicated that the percentages of pathogens with MICs of 0.5 mg/L or below for common multidrug-resistant bacteria, *Pseudomonas aeruginosa*, *Acinetobacter baumannii*, and *Klebsiella pneumoniae* were 8.3%, 27.9%, and 76.9%, respectively (European Committee on Antimicrobial Susceptibility Testing, 2022).

**FIGURE 1:**
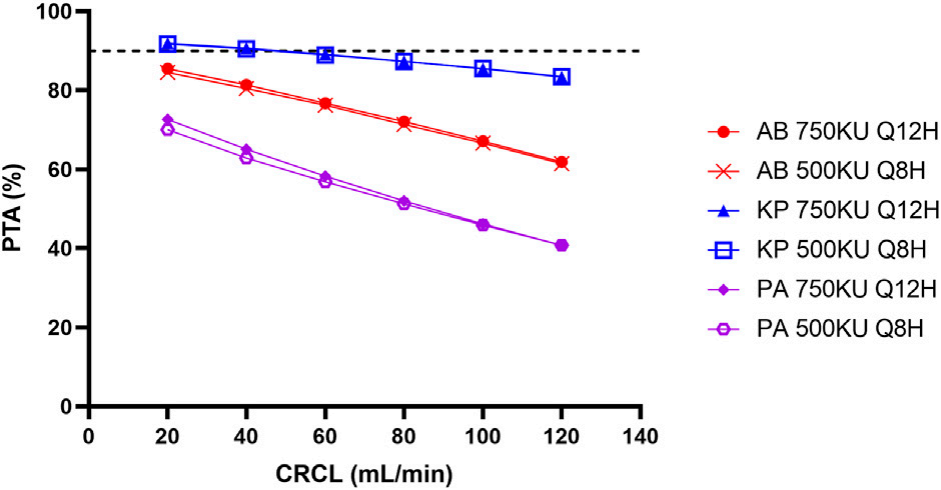
Simulated probability of achieving target attainment (PTA) of colistin sulfate at maximum daily dose for different micro-organisms. AB, *Acinetobacter baumannii*; KP, *Klebsiella pneumoniae*; PA, *Pseudomonas aeruginosa*; CLCR, creatinine clearance rate.

We have also run Monte Carlo simulation based on the published final model using NONMEM (version 7.5.0, ICON, Ellicott City, MD, United States) coupled with PDxPop (version 5.3, ICON, Gaithersburg, MD, United States). Virtual patients (10,000 patients per CLCR level) infected by bacteria with EUCAST MIC distribution were generated (European Committee on Antimicrobial Susceptibility Testing, 2022). The ratio of the unbound concentration–time curve to the MIC (*f*AUC/MIC) was also used as the PK/PD index, and the target *f*AUC/MIC was also set to 15 for *Pseudomonas aeruginosa* and *Acinetobacter baumannii*. The target *f*AUC/MIC for *Klebsiella pneumoniae* was set to 17.4, which was the target of polymyxin B for 1-log kill of bacteria in murine thigh infection model (Landersdorfer et al., 2018). AUCs at steady state of virtual patients were calculated using the pkr package (version 0.1.3) in R software (version 4.0.5) with a linear-up and linear-down method. The unbound fraction was the same as in the original study. The result is shown in Figure 1. The PTAs of the maximum daily dose (divided into 2 or 3 doses) were all below 90% for *Pseudomonas aeruginosa* and *Acinetobacter baumannii*. The MICs of *Klebsiella pneumoniae* were lower, but the PTAs achieved 90% only for patients with poor renal function. It should be noted that the PK/PD targets used in the simulation correspond to 1-log kill of bacteria in the animal model, but the targets for 2-log kill of bacteria are much higher (Tsuji et al., 2019). For some type of infection, such as lung infection, even the maximum tolerated dose cannot achieve bacterial stasis. Thus, from the perspective of current PK/PD data, intravenous colistin sulfate can only be used for infections caused by bacteria with very low MICs (e.g., 0.5 mg/L or below).

Moreover, Yu et al. stated that the PTA was higher for the dosing interval of 12 h than that of 8 h, at the same daily dose. This is not possible for an AUC-based PK/PD index and a PK model with linear elimination.

## Nephrotoxicity and clinical efficacy

Among the 42 included patients, only 2 patients developed acute kidney injury (AKI). Yu et al. claimed that the adverse events were not associated with colistin use but no detail was provided. Readers cannot judge whether those who experienced AKI were due to exposure to higher colistin concentration or due to other confounding factors. The prevalence of AKI in this study is quite low, as the reported prevalence of AKI in ICU patients was around 30% in other literatures (Chiofolo et al., 2019). The sample size of Yu et al.’s report was too limited to make any meaningful conclusions.

The 30-day mortality rate was 23.8% in Yu et al.’s study, and this result was satisfied in patients with drug-resistant bacterial infection. However, there may be no relationship between systemic colistin exposure and clinical efficacy. We noticed that nearly 70% of patients had respiratory tract infections, however, both preclinical and clinical studies indicated that intravenous polymyxins were suboptimal for lung infection (Cheah et al., 2015; Tsuji et al., 2019). The included patients also received other antibiotics (52.4% carbapenems, 47.6% cefoperazone-sulbactam, and other antibiotics) and inhaled colistin (52.4% of the patients), which may contribute to the low mortality, rather than intravenous colistin sulfate.

## Conclusion

A clear convention for the dose of colistin sulfate is urgently needed. PK/PD modeling showed that the PTA of colistin sulfate was poor for common hospital-acquired infections caused by *P. aeruginosa*, *A. baumannii*, and *K. pneumoniae*, even at the highest daily dose. Based on the very limited literature, intravenous colistin sulfate can only be used when the MIC of the pathogen is below 0.5 mg/L. The dose of colistin sulfate should be further optimized by balancing efficacy and toxicity.
